# Advances in the role of m^6^A RNA modification in cancer metabolic reprogramming

**DOI:** 10.1186/s13578-020-00479-z

**Published:** 2020-10-12

**Authors:** Xiu Han, Lin Wang, Qingzhen Han

**Affiliations:** Center of Clinical Laboratory, Suzhou Dushu Lake Public Hospital, 9#, Chongwen Road, Suzhou, 215000 People’s Republic of China

**Keywords:** N^6^-methyladenosine, Detection techniques, Cancer metabolic reprogramming, Glycolysis

## Abstract

N^6^-methyladenosine (m^6^A) modification is the most common internal modification of eukaryotic mRNA and is widely involved in many cellular processes, such as RNA transcription, splicing, nuclear transport, degradation, and translation. m^6^A has been shown to plays important roles in the initiation and progression of various cancers. The altered metabolic programming of cancer cells promotes their cell-autonomous proliferation and survival, leading to an indispensable hallmark of cancers. Accumulating evidence has demonstrated that this epigenetic modification exerts extensive effects on the cancer metabolic network by either directly regulating the expression of metabolic genes or modulating metabolism-associated signaling pathways. In this review, we summarized the regulatory mechanisms and biological functions of m^6^A and its role in cancer metabolic reprogramming.

## Introduction

N^6^-methyladenosine (m^6^A) is the most prevalent type of RNA modification of eukaryotic mRNAs [1, 2] and plays an important role in many biological functions including tissue development [3], naive pluripotency and stem cell differentiation [4], the heat shock response [5] and DNA damage [6]. m^6^A has been increasingly implicated in various human diseases such as obesity [7], diabetes [8], infertility [9], metabolic syndrome, and cancers [10–12]. In various cancers, m^6^A functions as a promoter or suppressor in cancers by regulating the expression of cancer-related genes, which may affect the initiation [13], proliferation [14], differentiation [15], metastasis [16] and metabolic reprogramming of cancer cells [17]. In 2011, He et al. discovered that fat mass and obesity-associated protein (FTO) exhibited efficient demethylation of m^6^A residues in RNA in vitro [18]. Based on the finding that the internal amino acid sequence of FTO was similar to the active domains of DNA demethylases, a second m^6^A demethylase, Alk B homolog 5 (ALKBH5), was identified and confirmed [19]. Since then, major insights into the biological functions and regulatory mechanisms of m^6^A have been reported. Although FTO was found to be more active on N6,2′-O-dimethyladenosine (m^6^Am; at the cap + 1 position) than on m^6^A in internal mRNA in experiments [20], the FTO-mediated demethylation events that act on internal m^6^A are more important. This finding is because the fact that total cap m^6^Am level is less than 1/20 of that of the internal m^6^A, and approximately 95% of the observed m^6^A increases occurred on internal sites when FTO was knocked down in AML cells. Moreover, Darnel and Ke et al. concluded that the methylation and demethylation of m^6^A were constrained in the nucleus and that no specific sites or demethylation of specific m^6^A residues were added to the mRNA in the cytoplasm [21]. Several groups have argued over the reversibility of m^6^A methylation in vivo [21, 22]. Currently, little is known about when the m^6^A actually occurs in the formation of cellular mRNA; hence, additional investigation is required. The field is still in its infancy. Thus, the possible function of the nuclear methylations and apparent demethylations requires further investigation.

Given that altered metabolism is a core hallmark of cancer, one of the recent research hotspots in the cancer field is metabolic reprogramming. This article summarizes the current understanding of m^6^A and cancer metabolic alterations, together with the crosstalk between m^6^A and nutrition, metabolism, and tumorigenesis. We further discuss whether m^6^A could be used in a therapeutic strategy targeting cancer metabolic reprogramming. These findings can be used to develop clinical guidelines and a novel therapeutic approach for cancer treatments involving early diagnosis, long-term follow-up, and prognosis.

## The regulatory mechanisms of m^6^A

**Fig. 1:**
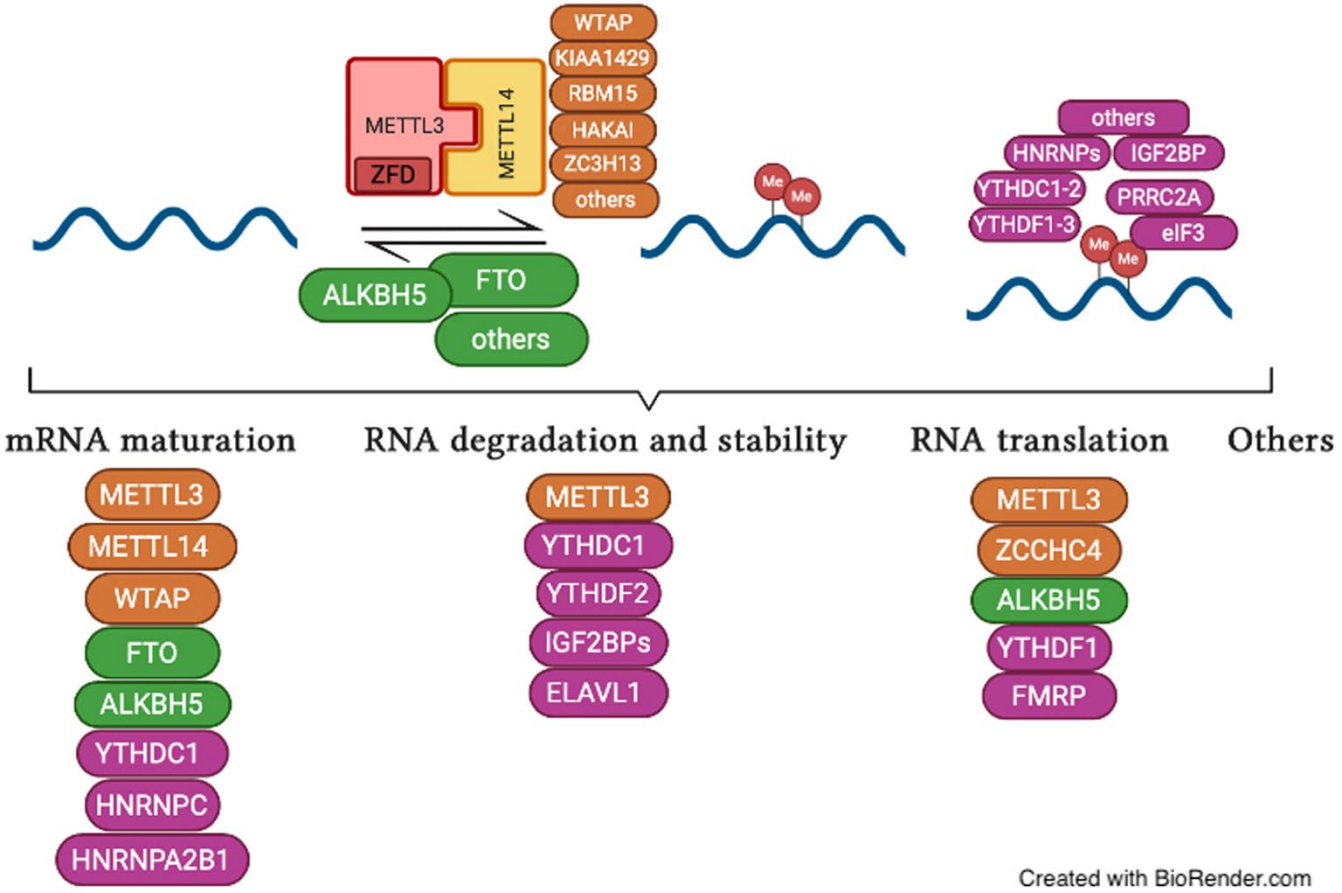
The process and molecular functions of m^6^A methylation. The effectors in m^6^A include ‘writers’, ‘readers’ and ‘erasers’. Writer methyltransferase installs m^6^A methylation on target RNAs via the methyl groups of S-adenosylmethionine (SAM) transferase. FTO and ALKBH5 were two major RNA demethylases that catalyze the removal of m^6^A on RNA in a Fe(II)/α-KG (α-ketoglutarate)-dependent manner. Methyltransferases and demethylases cooperate in modulating the distribution and abundance of m^6^A in RNAs, meanwhile the ‘readers’ specifically recognize and bind m^6^A-RNAs to control their fate and regulate downstream functions

m^6^A mainly occurs at the consensus motif DRACH (D corresponds to A, G or U; R corresponds to G or A; H corresponds to A, C or U) [23, 24], and it is enriched in the 5′-untranslated region (5′-UTR), 3′-untranslated region (3′-UTR) and coding DNA sequence (CDS) proximal to the stop codon of mRNAs [25, 26]. The effectors of m^6^A include ‘writers’, ‘readers’ and ‘erasers’ (Fig. 1). The writer methyltransferase adds m^6^A methylation on target RNAs via the methyl groups of S-adenosylmethionine (SAM) transferase [27]. The methyltransferase complex comprises the catalytic subunit methyltransferase like 3 (METTL3) and the catalytically inactive but structurally stabilizing subunit METTL14. The methyltransferase domains of METTL3 (MTD3, residues 357–580) and METTL14 (MTD14, residues 111–456) engage in extensive contact with each other to form a stable heterodimer, which, with the inclusion of the two Cys-Cys-Cys-His (CCCH)-type zinc-binding (ZFD) motifs of METTL3, can catalyze the addition of m^6^A [28, 29]. The ZFD serves as the target recognition domain for its special binding to the GGACH consensus sequence in the RNA [30, 31] and thus is responsible for the methyltransferase activity of the METTL3-METTL14 complex. For normal m^6^A modification to occur in cells, the METTL3-METTL14 complex also needs to associate with additional factors, such as tumor 1-associated protein (WTAP) [29, 32], KIAA1429 (also called Virilizer), RNA binding motif protein 15 (RBM15), the E3 ubiquitin ligase HAKAI, zinc finger CCCH domain-containing protein 13 (ZC3H13), and etc.[33–35].

However, FTO and ALKBH5 are two major RNA demethylases that catalyze the removal of m^6^A on RNA in an Fe(II)/α-KG (α-ketoglutarate)-dependent manner [36]. Structurally, ALKBH5 preferably demethylates m^6^A single-stranded RNA (ssRNA) over double-stranded DNA (dsDNA) because the loop (amino acids 229–243) causes a steric clash with the complementary strand of dsDNA. The m^6^A base is predicted to pack against His-204 and is in a pocket composed of Arg-130 and Tyr-139 in ALKBH5, which contributes to the m^6^A recognition [37]. In addition, the in vivo substrates of FTO include m^6^A and cap m^6^Am in mRNA, m^6^A and m^6^Am in snRNA, and m^1^A in tRNA. FTO exhibits a preference for the nucleobase m^6^Am over internal m^6^A in ssRNA, and the key residues (such as E234) in the catalytic pocket of FTO, rather than the structural differences of the ribose ring, function in nucleobase selection and recognition. The sequence and the tertiary structure of RNA affect the catalytic activity of FTO [38]. Methyltransferases and demethylases cooperate in modulating the distribution and abundance of m^6^A in RNAs, while the ‘readers’ specifically recognize and bind m^6^A-RNAs to control their fate and regulate downstream functions [39]. One class of direct and robust m^6^A readers are proteins containing the YT521-B homology (YTH) domain, including YTH domain family 1 − 3 (YTHDF1 − 3) [40] and YTH domain containing 1 − 2 (YTHDC1 − 2) in humans [41]. Other class of direct m^6^A binding proteins include the heterogeneous nuclear ribonucleoprotein A2B1( HNRNPA2B1) [42]; insulin-like growth factor 2 (IGF2) mRNA-binding proteins 1, 2 and 3 (IGF2BP1/2/3) [43], eukaryotic initiation factor 3 (eIF3) [44], and proline rich coiled-coil 2A (PRRC2A) [45]. In addition, there are other m^6^A readers such as HNRNPC [46] and HNRNPG [47, 48], which, rather than directly recognize the m6A group, accessibly bind the RNA-binding motifs upon m^6^A methylation of the RNA [48].

Overall, the installation, demethylation and recognition of m^6^A involved many effectors and pathways. Further studies need to be conducted to uncover new available factors in m^6^A modification with the goal of attaining a thorough understanding of this epigenetic modification.

## The biological functions of m^6^A

m^6^A is involved in many cellular RNA processes, including transcription, splicing, nuclear transport, degradation, and translation (Fig. 1 and Table 1).

**Table 1:** The biological functions of m^6^A

Functions	Regulators	Underlying mechanism	References
RNA maturation	METTL3, METTL14, WTAP	METTL3, METTL14, and WTAP all localize with pre-mRNA processing factors residing in the nuclear speckles	[116]
	FTO	The cellular FTO protein is present in a dot-like manner in nucleoplasm, and partially colocalizes with splicing or splicing-related speckle factors	[18]
	ALKBH5	ALKBH5 colocalizes well with mRNA-processing factors in nuclear speckles. SRPK1 translocate from nucleic locations to dot-like cytoplasmic sites, and ASF/SF2 switches from splicing factors to export adaptor proteins, promoting mRNA export, when depleting ALKBH5	[50]
	FTO	m^6^A was overrepresented in both alternative cassette exons and intron retention splicing events and peaks within cassette exons increased upon FTO depletion	[49]
	YTHDC1	YTHDC1 promotes exon inclusion of targeted mRNAs through facilitating SRSF3 while blocking SRSF10 mRNA binding	[53]
	HNRNPA2B1	HNRNPA2B1 binds to m^6^A containing sites on nuclear pri-miRNAs, and it interacts with the DGCR8 protein to facilitate the processing and maturation of pri-miRNAs	[42]
	HNRNPC	An m^6^A site in the lncRNA MALAT1 induces a local change in structure that increases the accessibility of a U5-tract for recognition and binding by HNRNPC	[46, 57]
RNA degradation	YTHDF2	YTHDF2 selectively binds to and destabilizes m^6^A-containing mRNA through direct recruitment of the CCR4-NOT deadenylase complex	[62]
	YTHDF2	P/Q/N-rich N terminus of YTHDF2 localizes the YTHDF2-m^6^A-mRNA complex to P bodies for committed degradation	[63]
	HuR	HuR interacted with SOX2 mRNA containing m^6^A to block the miRNA-dependent mRNA degradation and increase the stability	[66]
RNA translation	METTL3	mRNA nuclear export diminished when silencing METTL3	[68]
	ALKBH5	mRNA nuclear export accelerated when knocking down ALKBH5	[50]
	FMRP	FMRP promoted the nuclear export of methylated mRNAs in a CRM1-dependent way during neural differentiation	[69]
	YTHDF1	YTHDF1-mediated translation promotion increases translation efficiency, ensuring effective protein production from dynamic transcripts that are marked by m^6^A	[70]
		The presence of an m^6^A within a codon alters cognate tRNA selection to be kinetically unfavorable, with m^6^A acting as a barrier to tRNA accommodation and translation elongation	[71]
	ZCCHC4	ZCCHC4, a new m^6^A methyltransferase, catalyzed m^6^A4220 methylation in human 28S rRNA and also interacted with a subset of mRNAs, to affected global translation	[73, 74]

### m^6^A modulates mRNA maturation

Chemical inhibition of m^6^A formation caused changes in the ratio between precursor and mature mRNAs, and m^6^A was observed to be significantly enriched in both multi-isoform genes and alternatively spliced exons [49]. Immunofluorescence analysis showed that METTL3, METTL14, WTAP, FTO, and ALKBH5 colocalized well in nuclear speckles, the site where splicing factors accumulate [50]. These results suggest a potential role of m^6^A in mRNA splicing. A combination of transcriptome analyses and m^6^A-seq revealed that m^6^A is enriched in exonic regions flanking 5′- and 3′-splice sites, spatially overlapping with mRNA splicing regulatory serine/arginine-rich (SR) protein binding motifs. Modulating the expression of METTL3, ALKBH5 and FTO induces large-scale alterations in splicing patterns [23, 34, 49–51]. When ALKBH5 was depleted and the demethylation activity was diminished, serine-arginine protein kinase 1 (SRPK1) translocated from nuclear locations to dot-like cytoplasmic sites. Because SRPK1 is one of the main kinases responsible for the phosphorylation of alternative splicing factor/splicing factor 2 (ASF/SF2) [52], the depletion of ALKBH5 induced the ASF/SF2 switch from splicing factors to export adaptor proteins to promote mRNA export [50]. YTHDC1 promotes the exon inclusion of targeted mRNAs by facilitating serine and arginine rich splicing factor 3 (SRSF3) while blocking SRSF10 mRNA binding, demonstrating how YTHDC1 directly regulates mRNA splicing by bridging interactions of trans- and cis-regulatory elements [53]. In addition, HNRNPA2B1 binds to ‘RGm^6^AC’-containing sites on nuclear pri-miRNAs and interacts with the DGCR8 protein, a component of the pri-miRNA microprocessor complex, to facilitate the processing and maturation of pri-miRNAs [42].

However, m^6^A indirectly regulates mRNA splicing by modulating the structure of mRNA. m^6^A residues within RNA stems can destabilize the thermostability of model RNA duplexes without precluding Watson–Crick base pairing and make them more single-stranded or accessible [54–56], thus enhancing their interactions with HNRNPC. Consequently, m^6^A functions as an mRNA structure remodeler to affect mRNA maturation through interference with post-transcriptional regulator binding activities [57]. Taken together, these observations demonstrate that m^6^A modulates RNA maturation in direct and indirect ways.

### m^6^A modulates RNA degradation and stability

Degradation plays a fundamental role in maintaining cellular homeostasis, as both a surveillance mechanism eliminating aberrant mRNAs or during RNA processing generating mature transcripts [58]. The deadenylation-dependent decay pathway is used by most mRNAs in eukaryotes [59]. Deadenylation is triggered by deadenylases, including among others, the CCR4–NOT complex in mammals [60]. Additionally, there are three predominant forms of cotranslational mRNA surveillance: nonsense-mediated decay (NMD), no-go decay (NGD), and non-stop decay (NSD) [61]. m^6^A ‘readers’ proteins were widely reported to bind m^6^A-methylated mRNA and control RNA decay in an m^6^A methylation-dependent manner. The carboxyl terminal domain of YTHDF2 selectively binds to m^6^A-containing mRNA, while the amino-terminal domain recruits the CCR4-NOT complex through the SH domain of CNOT1, the scaffolding subunit of the CCR4-NOT complex. This recruitment is essential for the deadenylation of m^6^A-containing mRNAs by two deadenylase subunits, CAF1 and CCR [62]. Additionally, the P/Q/N-rich amino-terminus of YTHDF2 localizes the YTHDF2-m^6^A-mRNA complex to more specialized mRNA decay machinery (P bodies, etc.) for committed degradation [63]. Subsequent studies proved that CCR4-NOT deadenylation complex proteins were notable binding partners of all three DF proteins, indicating the idea that all DF paralogs had a common role in mRNA degradation [64]. In addition, m^6^A modification around the start codon of SRSFs is involved in degradation through METTL3 and YTHDC1 mediation of NMD, regulating the expression of SRSFs [65]. Mechanistically, in METTL3-KD cells, premature termination (i.e., stop) codons (PTCs) in the mRNAs of SRSFs occur by exon inclusion or skipping upon METTL3 depletion. These mRNAs with PTCs are subjected to NMD. Reduced expression of SRSFs induces alternative splicing isoform switches of related oncogenes, such as BCL-XS and NCOR2, promoting glioblastoma multiforme (GBM) growth and progression.

Nevertheless, other relevant proteins, such as insulin-like growth factor 2 mRNA-binding proteins (IGF2BPs) and ELAVL1 (also known as HUR), promote the stability and storage of target mRNAs under various physiological conditions and thus affect gene expression output. The mRNA decay of C-MYC, Fascin Actin-Bundling Protein 1(FSCN1), Thymidine Kinase 1(TK1), and Myristoylated alanine-rich protein kinase C substrate Like 1 (MARCKSL1) was accelerated upon knockdown of IGF2BPs in HepG2 cells. Moreover, the mRNA stabilizing function of IGF2BPs was supported by its cofactors HuR and matrin 3 (MATR3) [43]. HuR is a well-established RNA stabilizer protein that binds to the U-rich regions at the 3′-UTR of thousands of transcripts and blocks miRNA targeting. Visvanathan et al. demonstrated that HuR interacted with SOX2 mRNA containing m^6^A to block the miRNA-dependent mRNA degradation and therefore to increase the mRNA stability [66]. Another RNA immunoprecipitation (RIP) analysis indicated increased HuR binding at the IGFBP3 3′-UTR in METTL3 or METTL14 knockdown cells with decreased m^6^A levels, suggesting that demethylation accompanies HuR binding. Because the predicted motifs of m^6^A and HuR binding sites differ substantially and the endogenous m^6^A and HuR sites do not always colocalize, spatial constraints may control m^6^A and HuR binding [67]. Thus, m^6^A methylation has complex and sophisticated functions involving both stabilization and destabilization of RNAs. More investigations are needed to identify how to equilibrate and coordinate these two processes in mammalian cells.

### m^6^A modulates RNA translation

In addition to the control of mRNA degradation, regulation of translation is critical for managing the quantity and duration of gene expression in eukaryotic cells. mRNA nuclear export decreased when METTL3 was silenced [68] but accelerated upon ALKBH5 knockdown [50]. Furthermore, the fragile X mental retardation protein (FMRP), an m^6^A reader, promoted the nuclear export of methylated mRNAs in a CRM1-dependent manner during neural differentiation [69]. These results proved that m^6^A played an important role in RNA nuclear export, thus modulating mRNA translocation in humans. Wang et al. showed that YTHDF1 promoted the ribosome loading of mRNA and directly accelerated the translation initiation rate of target mRNAs in cells, possibly via the association of YTHDF1 with the translation initiation complex [70]. However, when Jaffrey and his team reanalyzed the previously published data from Wang et al. together with their own independent ribosome profiling datasets and performed new polysome fraction analysis, they claimed that none of the DF proteins (including YTHDF1, YTHDF2 and YTHDF3) directly promoted translation of m^6^A-mRNAs in Hela cells. Instead, their major function was to mediate mRNA degradation [64]. The inconsistent results may be due to bioinformatic and technical issues. m^6^A, however, affects mRNA translation through DF-independent mechanisms. m^6^A could impact 3′-UTR length, indirectly affecting translation [21]. Moreover, the m^6^A in the 5′-UTR recruits eIF3 to promote translation [44]. Although the presence of an m^6^A within a codon does not perturb canonical base-pairing in the final step of tRNA accumulation, the steric effects caused by decreased thermodynamic stability of modified A-U pairs can block the tRNA accommodation and translation elongation [71].rRNA with m^6^A in the mature ribosome has been implicated in the regulation and activity tuning of protein synthesis because it tends to localize in functionally important regions [72]. ZCCHC4, a new m^6^A methyltransferase, catalyzed m^6^A4220 methylation in human 28S rRNA to affect global translation activity, which is required for cell proliferation and tumor growth. It also interacts with a subset of mRNAs [73, 74]. Consequently, m^6^A affects nuclear export and translation by regulating the biological behaviors of mRNA, tRNA, and rRNA.

## Methods for detection of m^6^A methylation

**Fig. 2:**
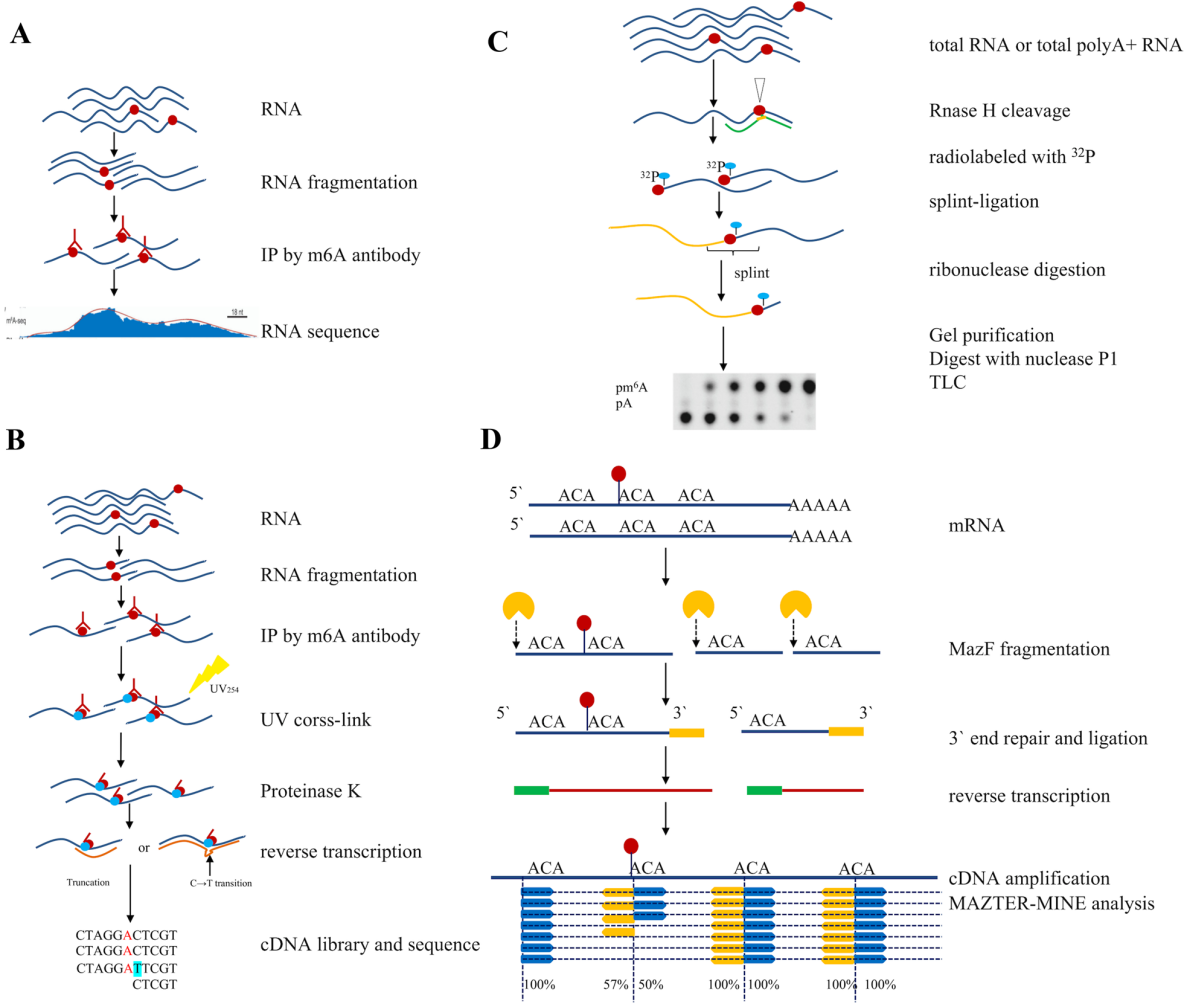
Methods for m^6^A methylation detection.** a** MeRIP-seq/m^6^A-seq; **b** Mapping m^6^A at individual-nucleotide resolution using crosslinking and immunoprecipitation: miCLIP and m6ACE-seq; **c** Site-specific cleavage and radioactive labeling followed by ligation-assisted extraction and thin-layer chromatography (SCARLET); **d** Antibody-independent m^6^A identification methods: (m6A-REF-seq) or MAZTER-seq

To date, most methods for global m^6^A detection have relied on immunoprecipitation of methylated RNAs using m^6^A-recognizing antibodies in a technique called methylated RNA immunoprecipitation sequencing (MeRIP-Seq/m^6^A-Seq) [75]. Although these methods have yielded unprecedented insights into the location and regulation of m^6^A in cellular RNAs, they have several limitations. Novel methods combined with multifield technologies have emerged (Fig. 2a–d), benefiting from the development of the high throughput sequencing and the liquid chromatography-tandem mass spectrometry (LC-MS/MS).

### MeRIP-seq/m^6^A-seq

MeRIP-seq/m^6^A-seq is by now the most extensively used molecular tool in m^6^A research. This technique uses anti-m^6^A antibodies to capture and enrich the m^6^A-containing RNA fragments (Fig. 2a), followed by high-throughput sequencing to profile m^6^A distributions in mammalian transcriptomes [76]. MeRIP-seq cannot identify the strict locations of the m^6^A in global transcriptomes because the resolution is 100–200 nt. Moreover, in the immunoprecipitation, large amounts of input RNA prevent global m^6^A detection in rare biological materials such as pathological tissues or early embryos [66]. Thus, novel methods are needed to unambiguously determine the m^6^A status at single-nucleotide resolution to further understand the biological function of this highly abundant modification.

### Mapping m^6^A at individual-nucleotide resolution using crosslinking and immunoprecipitation

In m^6^A individual-nucleotide resolution crosslinking and immunoprecipitation (miCLIP), extracted cellular RNA is cropped and crosslinked to an anti-m^6^A antibody using ultraviolet light (Fig. 2b). Antibody-crosslinked RNA fragments are then purified and converted into a cDNA library according to the PAR-CLIP protocol [77]. Then, crosslink-induced truncations or mutations after reverse transcription are investigated to identify exact m^6^A sites in the transcriptome. miCLIP detects m^6^A with high specificity and sensitivity [24]. Concurrently, using crosslinking and immunoprecipitation, m^6^A-CLIP [78] and m6A-crosslinking-exonucleasesequencing (m6ACE-seq) [79], different teams adopted techniques similar to miCLIP to map m6A at quantitative single-base resolution. m^6^A sites identified by m6ACE-seq exhibited significant overlap with sites identified by previous single-base resolution m^6^A-sequencing methods [79]. The sensitivity and specificity of m6ACE-seq were further validated by comparison with an orthogonal sequencing-independent single-base-resolution m6A mapping technique, site-specific cleavage and radioactive labeling followed by ligation-assisted extraction and thin-layer chromatography, known as SCARLET [80].

### Site-specific cleavage and radioactive labeling followed by ligation-assisted extraction and thin-layer chromatography

The sequencing methods only offer the distribution of m^6^A at a transcriptome-wide scale, but they cannot quantitatively detect the m^6^A fraction in the specific location, which are becoming increasingly important. SCARLET can accurately determine the m^6^A signature at single nucleotide resolution in any mRNA or lncRNA (Fig. 2c). Purification of the RNA of interest is not needed. Under the guidance of a complementary 2′-OMe/2′-H chimeric oligonucleotide, RNase H is applied to cut RNA, achieving site-specific cleavage 5′ to the candidate site. Before splint-ligating to a 116-nucleotide single-stranded DNA oligonucleotide using DNA ligase, the RNA fragment is radiolabeled using ^32^P. All RNA samples are treated with RNase T1/A for complete digestion except for the ^32^P-labeled candidate site. The 117/118-mers band on the denaturing electrophoresis gel is harvested and eluted and then digested by nuclease P1 into mononucleotides containing 5′ phosphate. Finally, thin-layer chromatography is performed to determine the m^6^A signature. SCARLET requires only common and readily available lab equipment and material, which makes it ready and available for researchers to investigate the dynamics and biology of RNA modification [80, 81].

### Antibody-independent m^6^A identification methods

Currently, most of the commonly used high-sensitivity LC-MS/MS and blotting methods are m^6^A antibody dependent, suffering from poor reproducibility and complicated processes. Additionally, it is difficult to quantify the level of methylation because of the affinity variation and batch effects of antibodies. Therefore, novel methods are still needed for whole transcriptome m^6^A identification and quantification to elucidate the dynamics and cellular functions of m^6^A in post-transcriptomic regulation. m^6^A-sensitive RNA-endoribonuclease-facilitated sequencing (m^6^A-REF-seq) [82] or MAZTER-seq [83] relies on the fact that MazF (Fig. 2d), an *Escherichia coli* toxin and RNA endoribonuclease, is sensitive to m^6^A modification in the ACA motif. This enzyme specifically identifies and cleaves the unmethylated ACA motifs while leaving methylated (m^6^A)CA motifs intact [84]. With MAZTER-MINE (https://github.com/SchwartzLab/mazter_mine), a computational pipeline, data from paired-end sequencing are analyzed to identify and quantify methylation sites following end repair, ligation, reverse transcription, and cDNA amplification [83]. Rapid and simplified experimental design without antibody-enrichment substantially reduces the starting RNA amount and sample preparation time, which addresses the limitations of current antibody-dependent methods. In addition, this method can capture the subtle changes of m^6^A during metabolic processes, highly advancing the dynamic studies of RNA m^6^A modification in different life stages.

## m^6^A methylation in cancer metabolic reprogramming

Reprogramming energy metabolism is one of the hallmarks of cancer, in addition to mutants, proliferative signaling, invasion and metastasis, and angiogenesis [85]. By taking advantage of the existing metabolic networks, cancer cells selectively activate or inhibit the metabolic pathways (e.g., aerobic glycolysis [86], disordered lipid metabolism [87], glutamine-dependent anaplerosis [88], and so on) (Table 2) to accelerate the proliferative capabilities. Thus, metabolite influx is altered and metabolites shunt into pathways that support biosynthesis to meet bioenergetic needs [89]. As the most abundant internal RNA modification, m^6^A plays an indispensable role in cancer metabolic reprogramming through either direct regulation of nutrient transporters and metabolic enzymes or indirect control of metabolic oncogenes and key components of metabolic pathways.

**Table 2:** m^6^A methylation in cancer metabolic reprogramming

Metabolism	Molecules	Underlying mechanism in metabolism	References
Glucose metabolism	HK2, GLUT1	m^6^A modification was closely correlated with glycolysis pathway activation in colorectal cancer patients’ tissues. Mechanically, HK2, and GLUT1 were found to be regulated by m^6^A modification and participate in glycolysis activation in colorectal cancer	[17]
	PKM2	FTO triggered the m^6^A demethylation of PKM2 mRNA and accelerated the translated production, thus promoting hepatocellular carcinoma tumorigenesis	[96]
	PIK3CB	A missense variant rs142933486 in PIK3CB reduced the PIK3CB m^6^A level and facilitated its mRNA and protein expression levels mediated by the m^6^A “writers” complex (METTL3/METTL14/WTAP) and YTHDF2	[97]
	EGFR, MEK/ERK signaling	YTHDF2 negatively modulated the EGFR mRNA stability in HCC via its binding the m^6^A site in the EGFR 3′-UTR, which in turn impaired the MEK/ERK pathway and consequently impedes the cell proliferation and growth	[102]
	NF-κB signaling	METTL3 positively regulated MYD88 expression through controlling m^6^A methylation status of MYD88-RNA, leading to the activation of NF-κB signaling	[103]
	NF-κB signaling	METTL3 activated NF-κB signaling by promoting the expression of IKBKB and RELA through regulating translational efficiency	[94]
	AKT signaling	m^6^A methylation normally attenuates AKT activity in the endometrium by promoting the m^6^Adependent translation of PHLPP2 and m^6^A-dependent degradation of transcripts encoding subunits of mTORC2, increasing proliferation and tumorigenicity in endometrial cancer	[14]
	AKT signaling	The association between m^6^A and AKT signaling was also confirmed in multiple tumor types including leukemia cells and clear cell renal cell carcinoma	[101, 117]
Lipid metabolism	ACC1, ACLY, DGAT2, EHHADH, FASN, FOXO, PGC1A, and SIRT1	ACC1, ACLY, DGAT2, EHHADH, FASN, FOXO, PGC1A, and SIRT1 were dramatically decreased in livers of hepatocyte-specific METTL3 knockout mice. CD36 and LDLR were also downregulated by improving the expression of FASN through its m^6^A demethylase activity	[106]
	SREBP1c, CIDEC	FTO increased lipid accumulation by a novel FTO/SREBP1c/CIDEC signaling pathway in an m^6^A-dependent manner in HepG2 cells	[108]
	SREBP1c, FASN, SCD1, ACC1	YTHDF2 could also bind to the mRNA of SREBP1c, FASN, SCD1, and ACC1, to decrease their mRNA stability and inhibit gene expression	[109]
	AMPK	m^6^A modification resulted in reduced AMPK activity	[110]
	FAM225A	m^6^A was highly enriched within FAM225A and enhanced its RNA stability	[111]
Glutamine metabolism	α-KG	FTO and ALKBH5 are α-KG-dependent dioxygenases and competitively inhibited by the structurally related metabolite D2-HG	[114, 115]

### m^6^A in glucose uptake and glycolysis

Aerobic glycolysis in cancer is activated under hypoxic conditions. Hypoxia could broadly increase the m^6^A of polyA + mRNA of certain genes that are closely associated with glycolysis (e.g., GLUT1 and MYC) [90, 91]. MYC induce the expression of glucose transporters and most glycolytic enzymes and can prominently drive aerobic glycolysis [92, 93]. The methyltransferase METTL3 upregulates MYC expression at multiple levels; for instance, METTL3 upregulates MYC mRNA stability by addition of methylation mainly around the stop codon and 3′-UTR [94], MYC mRNA elongation through AFF4 [95], and MYC transcription. Chen et al. found that m^6^A modification was directly correlated with activating the glycolytic pathway in colorectal cancer. Mechanistically, METTL3 increased the stability of HK2 and GLUT1 mRNA transcripts due to the m^6^A recognition of IGF2BP2/3. The METTL3-HK2/GLUT1-IGF2BP axis plays a critical role in the pathogenesis of colorectal cancer [17]. Moreover, FTO was shown to trigger the m^6^A demethylation of PKM2 mRNA and accelerated translation, leading to tumorigenesis of hepatocellular carcinoma [96].

In addition, m^6^A has broad effects on glycolysis-associated signaling pathways. A RIP assay with an antibody against m^6^A showed that the overexpression of PIK3CB containing the rs142933486-T allele (PIK3CB[T]) decreased the m^6^A level of PIK3CB compared with that of PIK3CB[G], indicating that the missense variant rs142933486 G > T in PIK3CB reduced the m^6^A level. Mechanistically, the variant is located 3 bp from a predicted m6A site. m^6^A is enriched at the consensus motif of DRACH; herein, D corresponds to A, G and U. Therefore, the G > T base change may disrupt the recognition by the m^6^A ‘writers’ complex (METTL3-METTL14-WTAP) and ‘erasers’, reducing m^6^A levels. However, m^6^A-methylated PIK3CB is recognized by YTHDF2, substantially decreasing the mRNA and protein expression by influencing its mRNA stability. The rs142933486 G > T in PIK3CB in turn enhances PIK3CB expression [97]. PIK3CB further activates the AKT pathway, whose downstream transcription factors can mediate glycolytic enzymes [98, 99]. Moreover, m^6^A methylation normally attenuates AKT activity in the endometrium by promoting the m^6^A-dependent translation of PHLPP2 and m^6^A-dependent degradation of transcripts encoding subunits of mTORC2, increasing the proliferation and tumorigenicity of endometrial cancer cells [14]. Other reported studies in multiple tumor types have also confirmed the association between m^6^A and AKT signaling, including that in leukemia cells [100] and clear cell renal cell carcinoma [101]. Notably, YTHDF2 functioned as a tumor suppressor in HCC by negatively modulating EGFR mRNA stability via its binding to the m^6^A site in the 3′-UTR of EGFR mRNA, in turn impairing the MEK/ERK pathway and consequently impeding the cell proliferation and growth [102]. METTL3 elimination inhibited the proteasome-mediated IκBα degradation and p65 phosphorylation, thereby restraining NF-κB nuclear translocation and leading to its transcriptional repression. Mechanistically, METTL3 installed m^6^A methylation on MYD88 mRNA to positively regulate MYD88 expression, allowing in the activation of NF-κB signaling [103]. METTL3 also activated NF-κB signaling by promoting the expression of IKBKB and RELA by regulating translational efficiency [94]. As we described above, the “writers”, “erasers” or “readers” induce m^6^A fluctuation of various mRNAs, indicating critical roles in glucose metabolism via glycolytic enzymes or associated signaling pathways.

### m^6^A in lipid metabolism

Increased de novo fatty acid synthesis and alternation of fatty acid uptake and catabolism elevate the rate of lipogenesis, allowing tumor cells to maintain their high proliferative rate. Lipid metabolic reprogramming allows cancers to adjust the metabolic demands toward the synthesis of macromolecules, the main lipids for the biogenesis of membranes and various signaling incentives to support tumorigenesis [104, 105]. ACC1, ACLY, DGAT2, EHHADH, FASN, FOXO, PGC1A, and SIRT1 are critical for the regulation of fatty acid synthesis and oxidation; however, they were dramatically decreased in the livers of mice with hepatocyte-specific METTL3 knockout. Additionally, the levels of two important regulators of cholesterol metabolism, CD36 and LDLR, were also downregulated in these mice due to the improvement in expression of FASN [106]. One recent study suggested that METTL3-mediated m^6^A modification led to LINC00958 upregulation by stabilizing its RNA transcript, which subsequently activated the miR-3619-5p/HDGF axis to facilitate lipogenesis in HCC. Key enzymes in lipogenesis, including SREBP1, FASN, SCD1, and ACC1, were also affected by LINC00958. These results delineated the m^6^A-involved regulatory mechanisms in lipogenesis of HCC [107]. Chen et al. showed that FTO increased lipid accumulation by a novel FTO/SREBP1c/CIDEC signaling pathway in an m^6^A-dependent manner in HepG2 cells and provided insight into the molecular mechanism of FTO in hepatic lipogenesis [108]. YTHDF2 could also bind to the mRNA of lipogenic genes, including SREBP1c, FASN, SCD1, and ACC1, to decrease their mRNA stability and inhibit gene expression [109].

In addition, m^6^A is closely associated with several signaling pathways to regulate lipid metabolism. m^6^A modification promoted the translation of protein phosphatase 1A, magnesium-dependent, alpha isoform (PPM1A), a negative AMPK regulator, but decreased the expression of calcium/calmodulin-dependent protein kinase kinase 2 (CAMKK2), a positive AMPK regulator, by reducing its RNA stability [110]. Thus, m^6^A modification resulted in reduced AMPK activity. AMPK affected PARK2 mRNA stability in a YTHDF2-dependent manner through FTO-dependent demethylation of m^6^A. Furthermore, m^6^A was highly enriched in FAM225A and enhanced its RNA stability [111]. In summary, m^6^A plays an important role in the lipid metabolic reprogramming of cancer.

### m^6^A in glutamine metabolism

Glutamine, as a source of carbon and nitrogen for biomass accumulation, participates in biosynthesis, energetics, and cellular homeostasis, reinforcing tumor growth and vitality [112]. Glutamine can be converted into α-KG to replenish the TCA cycle through two mechanisms: glutamate dehydrogenase (GLUD1) or transaminases [113]. FTO and ALKBH5 are α-KG-dependent dioxygenases and competitively inhibited by the structurally related metabolite D-2-hydorxyglutarate (D2-HG), which aberrantly accumulated in isocitrate dehydrogenase 1 or 2 (IDH1/2)-mutant tumors [114, 115]. Therefore, the effects of m^6^A on cancer pathogenesis need to be interpreted in the context of glutamine metabolism, but related studies are limited. Interactions between m^6^A and glutamine catalytic enzymes and signaling pathways remain to be explored.

## Conclusion and perspectives

Studies in the past few decades have shown that aberrant distribution and abundances of m^6^A drive tumorigenesis, at least in part through the control of cell metabolism [17, 96, 97]. Future studies aimed at refining our molecular map of the crucial regulatory nodes connecting m^6^A to the metabolic networks in different cancers will help reveal metabolic dependencies and novel therapeutic strategies. Due to the rapid development of technology methods for m^6^A, researchers have productively discovered many mechanisms of how m^6^A in cancer metabolism, including aerobic glycolysis, disordered lipid metabolism and glutamine-dependent anaplerosis. However, many challenges remain. The function of m^6^A in various cancers are still controversial. These functions are characterized by fluctuating distribution of m^6^A on different regions of mRNAs and subcellular readers responding to target genes that participate in different cellular processes. Moreover, with regard to the same target, the acceleration of m^6^A in the target could result in altered RNA splicing and increased translational capability, leading to upregulated mRNA. However, methylation at other loci may decrease the mRNA level because of enhanced m^6^A-dependent degradation. Given the extensive crosstalk among metabolic networks, it is particularly important to maintain homeostasis among various metabolic processes. Although there have been breakthroughs in the studies of glutamine metabolism in tumorigenesis and progression, the role of m^6^A may be less substantial.

In conclusion, elucidation of the molecular mechanisms underlying m^6^A in RNAs and its effects on cancer metabolic reprogramming could provide a better understanding of the epigenetics and abnormal metabolic characteristics of cancers. Additionally, these results may help predict cancer risk, achieve early diagnosis, track the prognosis of tumors fate, and ultimately provide novel therapeutic approaches.

## Data Availability

Not applicable.
